# The ROCK Inhibitor Y-27632 Improves Recovery of Human Embryonic Stem Cells after Fluorescence-Activated Cell Sorting with Multiple Cell Surface Markers

**DOI:** 10.1371/journal.pone.0012148

**Published:** 2010-08-13

**Authors:** Nil Emre, Jason G. Vidal, Jeanne Elia, Eric D. O'Connor, Rosanto I. Paramban, Michael P. Hefferan, Roman Navarro, Danielle S. Goldberg, Nissi M. Varki, Martin Marsala, Christian T. Carson

**Affiliations:** 1 BD Biosciences, La Jolla, California, United States of America; 2 Human Embryonic Stem Cell Core Facility, University of California San Diego, La Jolla, California, United States of America; 3 Department of Anesthesiology, School of Medicine, University of California San Diego, La Jolla, California, United States of America; 4 Department of Pathology, School of Medicine, University of California San Diego, La Jolla, California, United States of America; Katholieke Universiteit Leuven, Belgium

## Abstract

**Background:**

Due to the inherent sensitivity of human embryonic stem cells (hESCs) to manipulations, the recovery and survival of hESCs after fluorescence-activated cell sorting (FACS) can be low. Additionally, a well characterized and robust methodology for performing FACS on hESCs using multiple-cell surface markers has not been described. The p160-Rho-associated coiled kinase (ROCK) inhibitor, Y-27632, previously has been identified as enhancing survival of hESCs upon single-cell dissociation, as well as enhancing recovery from cryopreservation. Here we examined the application of Y-27632 to hESCs after FACS to improve survival in both feeder-dependent and feeder-independent growth conditions.

**Methodology/Principal Findings:**

HESCs were sorted using markers for SSEA-3, TRA-1-81, and SSEA-1. Cells were plated after sorting for 24 hours in either the presence or the absence of Y-27632. In both feeder-dependent and feeder-independent conditions, cell survival was greater when Y-27632 was applied to the hESCs after sort. Specifically, treatment of cells with Y-27632 improved post-sort recovery up to four fold. To determine the long-term effects of sorting with and without the application of Y-27632, hESCs were further analyzed. Specifically, hESCs sorted with and without the addition of Y-27632 retained normal morphology, expressed hESC-specific markers as measured by immunocytochemistry and flow cytometry, and maintained a stable karyotype. In addition, the hESCs could differentiate into three germ layers *in vitro* and *in vivo* in both feeder-dependent and feeder-independent growth conditions.

**Conclusions/Significance:**

The application of Y-27632 to hESCs after cell sorting improves cell recovery with no observed effect on pluripotency, and enables the consistent recovery of hESCs by FACS using multiple surface markers. This improved methodology for cell sorting of hESCs will aid many applications such as removal of hESCs from secondary cell types, identification and isolation of stem cell subpopulations, and generation of single cell clones. Finally, these results demonstrate an additional application of ROCK inhibition to hESC research.

## Introduction

HESCs have the unique ability to self-renew and give rise to ectodermal, mesodermal, and endodermal lineages [1], [2]. This capacity to differentiate into cells of all three germ layers provides an excellent system to study human development and model disease states. Additionally, as hESCs are a continuously self-replicating population of cells, they have the potential to be a stable source of numerous cell types for regenerative medicine. Unfortunately, hESCs are sensitive to even the most routine manipulations, such as passaging and cryopreservation, illustrating the need for technical advancements to realize their full potential [3], [4].

Cell dissociation induced apopotosis has been attributed to the inherent sensitivity of hESCs and has duly received much attention. A significant breakthrough for ameliorating this problem was the identification of Y-27632, a selective inhibitor of the p160-Rho-associated coiled kinase (ROCK) [5], [6], as a factor that enhanced hESC survival upon single cell dissociation [7]. Subsequently, Y-27632 has been used in various applications in stem cell research where extensive cell death occurs. The post-thaw survival rate was enhanced by the addition of Y-27632 to hESCs grown in feeder-dependent and independent conditions and feeder-independent human induced pluripotent stem cells (hiPSCs) [8]–[12]. Improved recovery from cryopreservation was also reported from the addition of Y-27632 to other stem cell types including non-human primate embryonic stem cells [13] and bone marrow-derived mesenchymal stem cells [14]. Inhibition of ROCK also improved the survival upon dissociation of hESC-derived cardiomyocyte and non-cardiomyocyte cells [15]. The recovery upon dissociation as well as the transplantation of neural precursor cells derived from mouse embryonic stem cells was positively impacted by the addition of Y-27632 [16]. During differentiation, Y-27632 was applied to cells to improve survival upon differentiation of hESCs to retinal cells [17]. In reprogramming, Y-27632 has been used after viral transduction at culture media exchange to aid in the establishment of hiPSCs [18]. Y-27632 has also been applied to improve survival of additional cell types such as endothelial cells [19]and retinal ganglion cells [20]. All together, this body of research has demonstrated the utility and safety of Y-27632 for a variety of applications and cell types.

The utilization of cell surface markers, including SSEA-3, SSEA-4, TRA-1-60, TRA-1-81 and SSEA-1 to characterize hESCs is widely accepted [21], [22]. The ability to use these and other surface markers in combination with FACS to consistently isolate hESCs and their differentiated progeny would facilitate many applications. These applications include the removal of contaminating secondary cell types such as feeders, hESC-derived fibroblasts and spontaneously differentiating cells, as well as the identification and isolation of pure subpopulations, genetically modified cells, and single-cell clones. Thus far, the reported use of FACS to isolate and consistently recover hESCs has been limited and variable, necessitating the generation of robust and standardized sorting methods. HESCs have been sorted by the use of light scatter gating [23] and by the expression of GFP in genetically altered hESCs lines [24], [25]. A combination of sorting using a fluorescent reporter and SSEA-3 labeling of hESCs with the successful recovery of cells has also been reported [26]. Interestingly, the only study to date that used more than one endogenously expressed cell surface marker (SSEA-4 and TRA-1-81) to sort hESCs resulted in an inability to recover viable cultures post-sort [27]. All the previously mentioned studies that successfully sort hESCs report low recovery. Although a low recovery is to be expected since sorting of hESCs requires that the cells be dissociated to a single cell state, it also highlights the necessity for an improvement in sorting conditions for hESCs.

Since previous studies have shown that the addition of Y-27632 improves the survival of hESCs that have been dissociated to single cells (for review see [28]), we examined whether Y-27632 would improve the recovery of hESCs after sorting. Specifically, we describe that Y-27632 improved the recovery upon sorting of hESCs using three cell surface markers, SSEA-3 and TRA-1-81 for pluripotency and SSEA-1 to exclude spontaneously differentiating cells in the culture. Cell sorting could be performed on cells grown in feeder-dependent and feeder-independent growth conditions. After long-term culturing, sorted cells expressed markers for pluripotency, differentiated *in vitro* and *in vivo* to all three germ layers, and maintained a stable karyotype.

## Materials and Methods

### HESC Culture

For feeder-dependent growth conditions, H9 and H7 (WiCell) hESCs were grown on irradiated mouse embryonic fibroblast feeder cells in hESC feeder-dependent medium (DMEM-F12, 20% Knockout Serum Replacement, 0.1 mM nonessential amino acids (all Life Technologies), 2 mM glutamine (Hyclone), 0.1 mM β-mercaptoethanol (Millipore), and 10 ng/mL recombinant human bFGF (BD Biosciences)). Cells were passaged using 0.1 mg/ml collagenase IV (Life Technologies). For feeder-independent growth conditions, hESCs cells (H9 and HUES-9) were cultured on Falcon plates (BD Biosciences) coated with Matrigel or Matrigel hESC-qualified Matrix (BD Biosciences) in mTeSR1 maintenance media (Stem Cell Technologies) according to manufacturers' specifications. Feeder-independent hESCs were passaged with 1 mg/ml dispase (BD Biosciences).

### Cell Sorting and Recovery

HESCs were dissociated with Accutase (Innovative Cell Technologies) and stained using the Human Pluripotent Stem Cell Sorting and Analysis Kit (BD Biosciences) according to manufacturers' directions. Briefly, dissociated cells were passed through a 70-µm cell strainer (BD Biosciences), treated at room temperature for 10–15 minutes with 100 units/ml DNase (Sigma), and stained using FITC anti-SSEA-1, PE anti-SSEA-3, and Alexa Fluor 647 anti-TRA-1-81 on ice in hESC media containing 5 mM EDTA (Life Technologies). Cells were subsequently washed in media containing 5 mM EDTA and resuspended in media containing 5 mM EDTA at a concentration of 5×10^6^ cells/mL. Stained hESCs were sorted on a BD FACSAria II cell sorter at approximately 20 PSI using a 100-micron nozzle at an average acquisition rate of 3,000 cells per second into either feeder-dependent media or mTeSR1. Cells were washed twice in the appropriate hESC culture media prior to plating at a density of 1.2–2×10^6^ cells/well of a 6-well dish. Penicillin (100 units/ml)/streptomycin (100 µg/ml) (Lonza) was added to the hESC cultures for two weeks after sorting. Y-27632 (Calbiochem) was added to the hESC media at a concentration of 10 µM at the time of plating after sorting and removed from the media 24 hours after the sort. For viability studies, cells were sorted as above or passaged with Accutase and plated onto a 24-well dish at a density of 4.2×10^5^ cells/well. Cells were counted 24 hours after plating using a Vi-CELL analyzer (Beckman Coulter). For pre-treatment studies, cells were pre-treated with 10 µM Y-27632 for one hour prior to sort and subsequently sorted as described above. Post-sort, cells were plated onto a 24-well dish at a density of 2.5×10^5^ cells/well, treated with varying concentrations of Y-27632 (0–50 µM ), and counted 24 hours after plating.

### hESC Differentiation

To generate embryoid bodies and to spontaneously differentiate cells to ectodermal and mesodermal lineages, hESCs were scraped and placed in hESC feeder-dependent media minus bFGF or in DMEM-F12 (Life Technologies), 20% FBS (HyClone), 0.1 mM nonessential amino acids (Life Technologies), 2 mM glutamine (Hyclone), and 0.1 mM β-mercaptoethanol (Millipore) into low adhesion plates (Costar) for a period of 7–10 days. Embryoid bodies were subsequently placed onto 0.1% gelatin (Millipore) coated dishes for an additional 4–7 days. For directed differentiation to endoderm, hESCs were treated with 100 ng/mL Activin A (R&D Systems) in low FBS media for three days [29].

### Cell Characterization

Cells were grown on Falcon 96-well bioimaging plates (BD Biosciences), washed with 1× PBS, and fixed with BD Cytofix. Cells were stained, or permeabilized with 0.1% Triton (Sigma Aldrich) or BD Phosflow Perm Buffer III and stained.

Cells were stained with the following antibodies for immunofluorescence: Alexa Fluor 555 anti-Tra-1-81 (560123), Alexa Fluor 647 anti-Tra-1-60 (560122), Alexa Fluor 488 anti-SSEA-1 (560172), Alexa Fluor 555 anti-SSEA-4 (560218), Alexa Fluor 647 anti-SSEA-1 (560120), Alexa Fluor 488 anti-Oct-3/4 (560217), Alexa Fluor 647 anti-Sox2 (560294), anti-human Nanog (560109), Alexa Fluor 488 anti-GATA4 (560332), anti-Desmin (550626), Alexa Fluor 647 anti-Nestin (560393), anti-Sox1 (560749), and anti-FoxA2 (all BD Biosciences), anti-Sox17 (R&D Systems AF1924) and anti-Cardiac Troponin I (cTnI) (Millipore MAB1691). Purified antibodies were detected using Alexa Fluor 555 and 488 (Life Technologies) secondary antibodies. After antibody staining, the cell nuclei were counterstained using DAPI (Sigma Aldrich) or Hoechst (Sigma Aldrich). Cells were imaged on a Pathway™ 435 bioimager (BD Biosciences) using a 10× objective and acquired using BD Attovision Software.

FACS analysis was performed using a Human Pluripotent Stem Cell Sorting and Analysis Kit, a Human and Mouse Pluripotent Stem Cell Analysis Kit, and a Human Pluripotent Stem Cell Transcription Factor Analysis Kit (all BD Biosciences). Stained cells were analyzed on a BD LSR II flow cytometry system using FACSDiva Software (BD Biosciences).

For *in vivo* spinal grafting and identification of teratomas, adult athymic nude (rnu-/rnu-) male rats (320–350 g; n = 3 per group) were anesthetized with isoflurane (1.5–2% maintenance; in room air), placed into a spinal unit apparatus (Stoelting) and a partial Th12-L1 laminectomy performed using a dental drill (exposing the dorsal surface of L2–L5 segments). Using a glass capillary (tip diameter 80–100 µm) connected to a microinjector (Kopf Instruments), rats received spinal cell injections (0.5 µl/10,000–15,000 cell/injection) of hESC lines delivered in DMEF/F12 media. The duration of each injection was 60 s followed by 30 s pause before capillary withdrawal. The center of the injection was targeted into the central gray matter (distance from the dorsal surface of the spinal cord at L3 level approximately: 0.7–0.9 mm) [30]. Ten injections (500–800 µm rostrocaudally apart) were made on each side of the lumbar spinal cord. After injections, the incision was cleaned with penicillin-streptomycin solution and sutured in two layers. Three or four weeks after cell grafting, rats were deeply anesthetized with pentobarbital and phenytoin and transcardially perfused with 200 ml of heparinized saline followed by 250 ml of 4% paraformaldehyde in PBS. The spinal cords were dissected and postfixed in 4% formaldehyde in PBS overnight at 4°C. Paraffin-embeded sections (5 µm thick) were then cut and stained with H&E. Slides were analyzed with Leica (DMLB) microscope, images captured with Hamamatsu firewire camera and processed by Adobe Photoshop 7.0 (Adobe Systems).

Karyotyping service was provided by Cell Line Genetics (Madison, WI).

## Results

### Sorting strategy for hESCs

**Figure 1:**
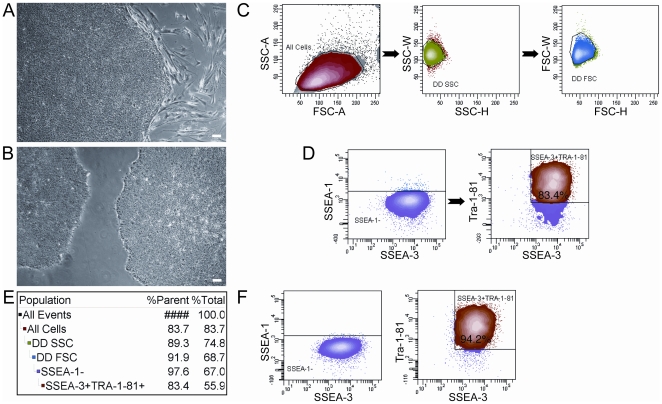
Sorting strategy for hESCs. Bright field images of **A**) H9 hESCs grown on feeders at passage 42 prior to sorting and **B**) H9 hESCs grown feeder-free at passage 33 prior to sorting. Cells were stained with SSEA-1, SSEA-3, and TRA-1-81 fluorochrome-conjugated antibodies. **C**) Single cells were first identified by light scatter gating, **D**) SSEA-1^−^ cells were gated out and SSEA-3^+^TRA-1-81^+^ cells were gated and then sorted. **E**) Gating hierarchy and **F**) post-sort purity is shown. Scale bars in (**A**) and (**B**) are 100 µm.

HESCs (H9) were grown in feeder-dependent (Fig. 1A) and feeder-independent (Fig.1B) conditions and routinely passaged in clusters with either collagenase IV or dispase, respectively. For sorting, Accutase was chosen to disassociate hESCs given that it was reported to dissociate hESCs in sorting for GFP positive cells [31]. For both feeder-dependent and feeder-independent (data not shown) sorting experiments, single cells of hESCs were first identified (Fig. 1C), and SSEA-1^−^/SSEA-3^+^/Tra-1-81^+^ cells were sorted from cultures (Fig. 1D). The percentage of hESCs that were SSEA-1^−^/SSEA-3^+^/Tra-1-81^+^ pre-sort (83.4%) (Fig. 1D–E) and post-sort (94.2%) (Fig. 1F) were compared to show enrichment for pluripotent hESCs after sorting.

### Y-27632 improves recovery upon sorting

**Figure 2:**
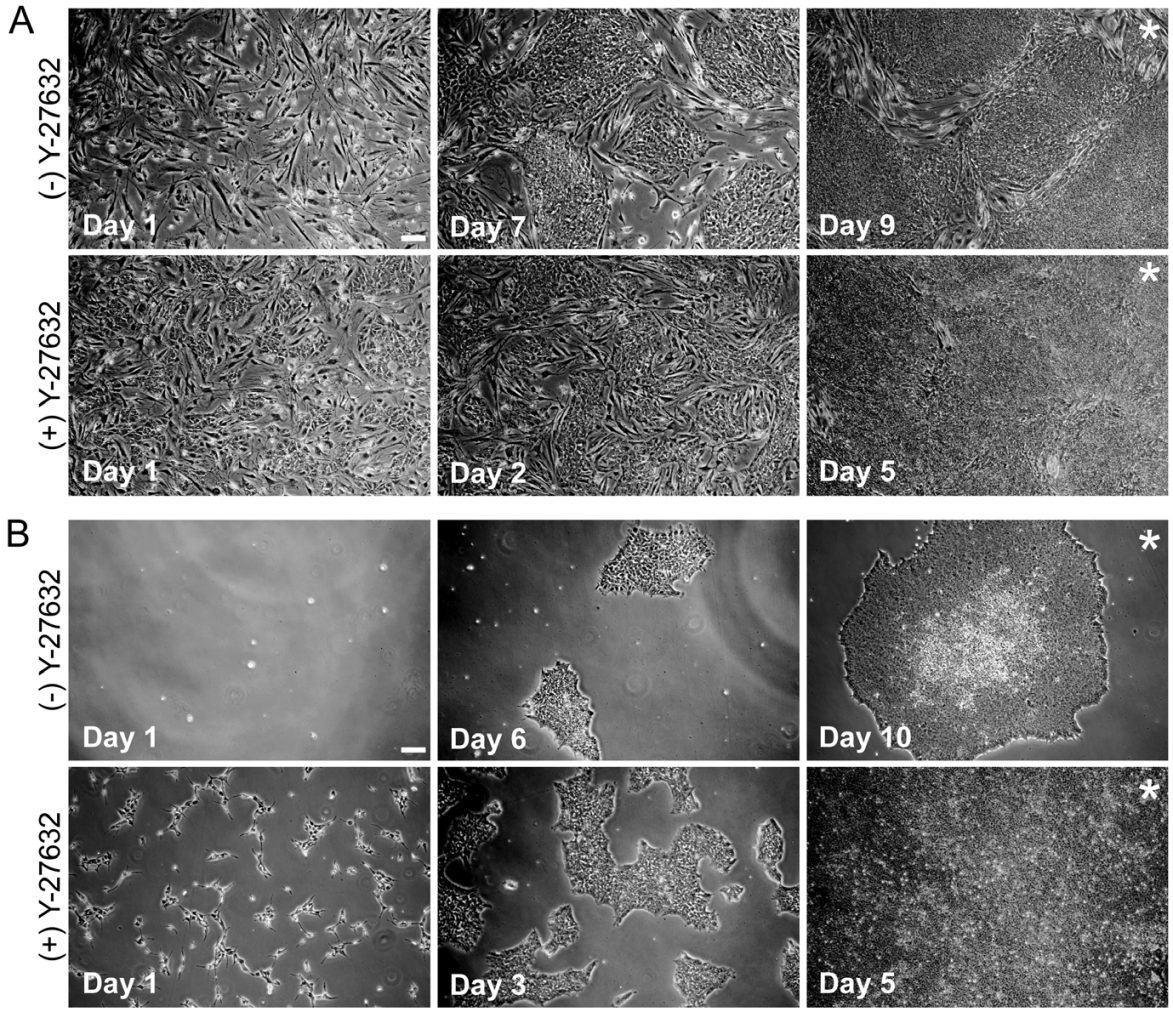
Morphology of hESCs after sorting without and with application of Y-27632. Bright field images of H9 hESCs at various days after sorting without and with Y-27632. **A**) HESCs grown on feeders (cells were passage 42 prior to sorting) **B**) HESCs grown feeder-free (cells were passage 33 prior to sorting). (*) indicates the day at which cells were passaged after the sort. Scale bars are 100 µm.

Although we were able to routinely recover hESCs by sorting using cell surface markers, the number of hESCs colonies that appeared after sorting was low and the colonies took longer to appear (as compared to passaging) in both feeder-dependent and feeder-independent conditions. We observed that plating cells at a higher density post-sort aided in recovery (data not shown). To further improve post-sort recovery, we applied Y-27632 to hESCs post-sort. We morphologically compared hESCs grown in feeder-dependent conditions that were treated with Y-27632 for 24 hours after sort to hESCs that received no treatment (Fig. 2A). Cells that received Y-27632 after sorting were visible upon day 1 post-sort and cells were ready to be passaged day 5 post-sort, whereas the untreated group was not ready to be passaged until day 9 post-sort. Similar trends were seen in hESCs grown in feeder-independent conditions where the addition of Y-27632 improved post-sort recovery and time to passage (Fig. 2B). Additionally, after Y-27632 treatment, cells had a more flattened morphology and eventually formed a monolayer in culture (Figure 2); this morphology has been previously reported upon application of Y-27632 after passaging and after cryopreservation [9], [31]. SSEA-1^−^/SSEA-3^+^/Tra-1-81^+^ H7 hESCs were also sorted several times but colonies only appeared in cultures that received Y-27632 post-sort (Fig. S1). H7 hESCs were not passaged until day 11 post-sort, comparable to H9 hESCs that were sorted without Y-27632.

**Figure 3:**
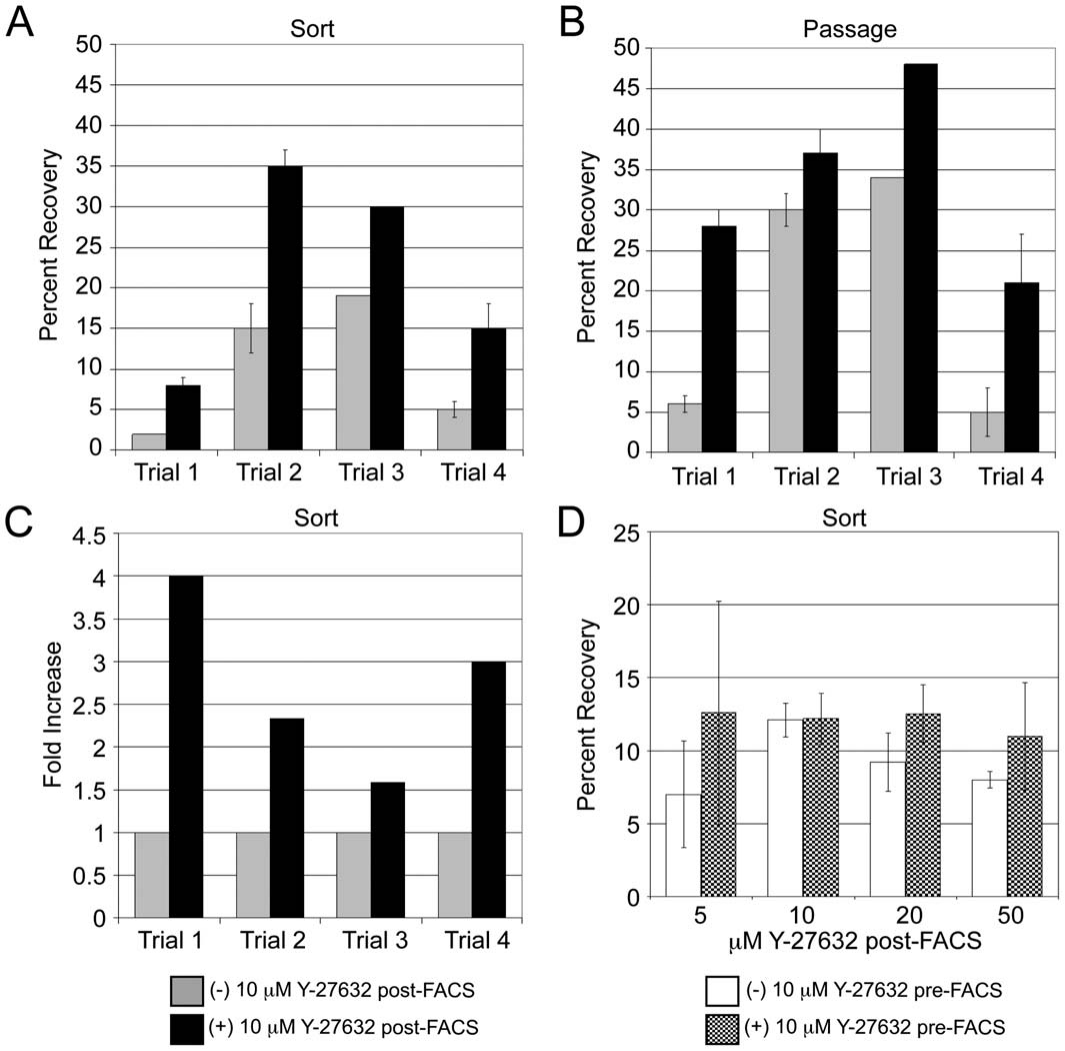
Recovery of hESCs grown feeder-free after sorting and passaging without and with Y-27632. Percent recovery of H9 hESCs at day 1 after **A**) sorting or **B**) passaging with Accutase. Percent recovery equals the number of cells recovered at day 1 after passage or sorting divided by the number of cells plated. A total of two (Trial 3) to three wells (Trials 1, 2 and 4) were counted for each trial group with error bars representing standard deviation. **C**) Fold increase in the recovery of cells at day 1 upon addition of Y-27632 after sorting. The number of cells recovered without Y-27632 was set at 1. **D**) Percent recovery at day 1 post-sort after pre-treatment of cells with Y-27632 and Y-27632 post-sort titration. A total of three wells were counted for each trial group with error bars representing standard deviation.

To quantitatively assess the impact of Y-27632 on cells during sorting and during routine passaging, hESCs grown in feeder-independent conditions were counted at day 1 post-sort and day 1 post-passage. We compared the percent recovery (number of cells recovered at day 1/number of cells plated) of hESCs that were plated with and without Y-27632 and either sorted (Fig. 3A) or passaged as single cells (Fig. 3B). For sorted cells, 8–35% of hESCs were recovered in the presence of Y-27632 as compared to only 2–19% without Y-27632 (Fig. 3A). Post-passaging, 21–48% of cells were recovered in the presence of Y-27632 versus 5–34% of cells without Y-27632 (Fig. 3B). The improved recovery of hESCs by using Y-27632 after passaging correlates with previous reports [9]. Not surprisingly, due to the increased manipulations involved in cell sorting, cells that were sorted had a lower percent recovery than cells that were passaged. The relative fold increase in survival after sorting and subsequent application of Y-27632 was compared between different sorts (Fig. 3C). Application of Y-27632 after cell sorting resulted in a 1.6 to 4 fold increase in survival compared to sorting without application of Y-27632.

To further define sorting conditions, we assessed whether pre-treatment of cells with Y-27632 would have an effect on post-sort recovery. There was no effect when H9 hESCs grown in feeder-independent conditions were pre-treated with Y-27632 prior to sorting (Fig. 3D) and this trend was observed in multiple pre-treatment experiments (data not shown). Additionally, the concentration of Y-27632 was varied post-sort to see if there was a dose-dependant impact on survival. In H9 hESCs, while using less than 10 µM Y-27632 had a negative impact on recovery if no-pretreatment was performed, there was no dose-dependent effect on survival at higher concentrations of Y-27632 (Fig. 3D). As previously reported, pre-treatment of hESC with Y-27632 resulted in a longer and more laborious cell dissociation [9]. In some cases we observed up to a 20% decrease in the initial cell population for sorting if cells were pre-treated with Y-27632 (data not shown). We also performed pre-treatment and concentration titrations of Y-27632 on the hESCs cell line HUES-9, to see if trends were similar. For two of three trials, there was no additive effect on survival after pre-treatment (Fig. S2B–C). For one trial (Fig. S2A), there was a positive impact of pre-treatment on survival. We cannot explain this discrepancy other than inherent variability in cell preparation during sorting or passaging. For all three trials, there was no substantial increase in survival at higher doses of Y-27632. All together, these data suggest that treatment with 10 µM Y-27632 post-sort is sufficient to improve cell survival. Interestingly, we consistently achieved a higher percent recovery with HUES-9 (30–55%) than with H9 (8–35%). Moreover, we were not able to reproduce pre-treatment experiments faithfully with the H7 line due to the sporadic and variable nature of these cells to recover post-sort. All together, these data highlight differences in the robustness of hESC lines in recovering from single cell dissociation and cell sorting.

### HESCs sorted with three surface markers and recovered with Y-27632 maintain pluripotency and a stable karyotype

**Figure 4:**
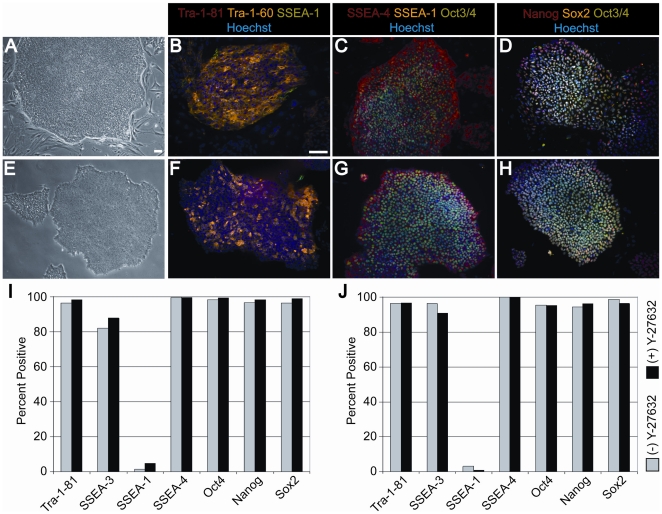
Characterization of hESCs upon extended passaging after sorting with application of Y-27632. **A**) Bright field image of H9 hESCs cultured on feeders at passage 26 post-sort and day 4 after passaging. **B–D**) Immunofluorescence labeling of hESCs cultured in feeder-growth conditions at passage 26 post-sort. **E**) Bright field image of hESCs cultured in feeder-free growth conditions at passage 10 post-sort and day 3 after passaging. **F–H**) Immunofluorescence labeling of hESCs in feeder-free growth conditions at passage 10 post-sort. Post-sort marker expression of H9 hESCs as measured by flow cytometry of **I**) Passage 14 post-sort on feeders with Y-27632 and passage 13 post-sort without Y-27632 and **J**) Passage 13 post-sort feeder-free hESCs with and without Y-27632. The percent positive expression from flow cytometry experiments after extended passage post-sort with and without Y-27632 is shown (data from flow analysis experiments is shown in Fig. S4). Scale bars in (**A–H**) are 100 µm.

HESCs sorted using cell surface markers and treated with Y-27632 after sorting were cultured for an extended period of time and subsequently characterized. Sorted cells that were grown in feeder-dependent conditions (Fig. 4A) and feeder-independent conditions (Fig. 4E) maintained typical hESCs morphology of compact colonies with distinct borders. Additionally, cells expressed various pluripotency markers by immunofluorescence (TRA-1-81, TRA-1-60, SSEA-3, Oct4, Nanog, and Sox2) and did not express the differentiation marker SSEA-1 (Fig. 4B–D, F–H). Similar morphology and marker expression was seen for cells that were sorted without Y-27632 in feeder-dependent and feeder-independent conditions (Fig. S3). To quantitatively assess expression of pluripotency markers, we analyzed the cells by flow cytometry (Fig. 4I–J; Fig. S4). Cells sorted in both feeder-dependent (Fig. 4I) and feeder-independent (Fig. 4J) conditions showed high levels of pluripotency markers. Cells that were sorted with and without Y-27632 both expressed high levels of pluripotency markers, indicating that cells maintained pluripotency after cell sorting. No differences were observed in the expression of pluripotency markers in cells that were sorted with and without Y-27632.

**Figure 5:**
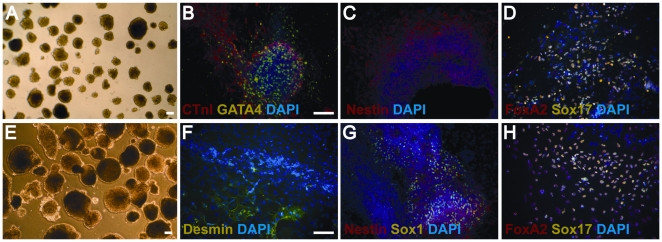
*In vitro* differentiative capacity of hESCs upon extended passaging after sorting with Y-27632. Bright field images of **A**) day 5 embryoid bodies from hESCs on feeders at passage 13 post-sort and **E**) day 6 embryoid bodies from hESCs feeder-free at passage 12 post-sort. Immunofluorescence of differentiated hESCs shows labeling for mesoderm (**B, F**) (GATA4 and cTnI and Desmin), ectoderm (**C, G**) (Nestin and Sox1), and endoderm (**D, H**) (FoxA2, Sox17). For feeder conditions (**B–D**), hESCs are at passage 14 post-sort (**B**); passage 13 post-sort (**C**); and passage 16 post-sort (**D**). For feeder-free conditions (**F–H**), hESCs are at passage 12 post-sort (**F**); passage 12 post-sort (**G**); and passage 13 post-sort (**H**). Scale bars are 100 µm.

Sorted hESC were also examined for their *in vitro* differentiative capacity. HESCs sorted with Y-27632 and grown in feeder-dependent (Fig. 5A) and feeder-independent conditions (Fig. 5E) were able to form embryoid bodies. Additionally, cells were able to differentiate into cell types that stained positive for mesoderm (GATA4, cTNI, Desmin) (Fig. 5B and 5F), ectoderm (Nestin, Sox1) (Fig. 5C and 5G), and endoderm (FoxA2, Sox17) (Fig. 5D and 5H). Cells that were sorted without the presence of Y-27632 were also able to differentiate into all three germ layers (Fig. S5). Sorted hESCs that were treated with and without Y-27632 had similar abilities to differentiate to all three germ lineages. Interestingly, we noticed that cells grown in feeder-independent conditions formed fewer beating cardiomyocytes. We were unable to obtain any beating cardiomyocytes in feeder-independent hESCs sorted with Y-27632 but the cells were able to differentiate to a mesodermal lineage as evidenced by the expression of desmin (Fig. 5F).

**Figure 6:**
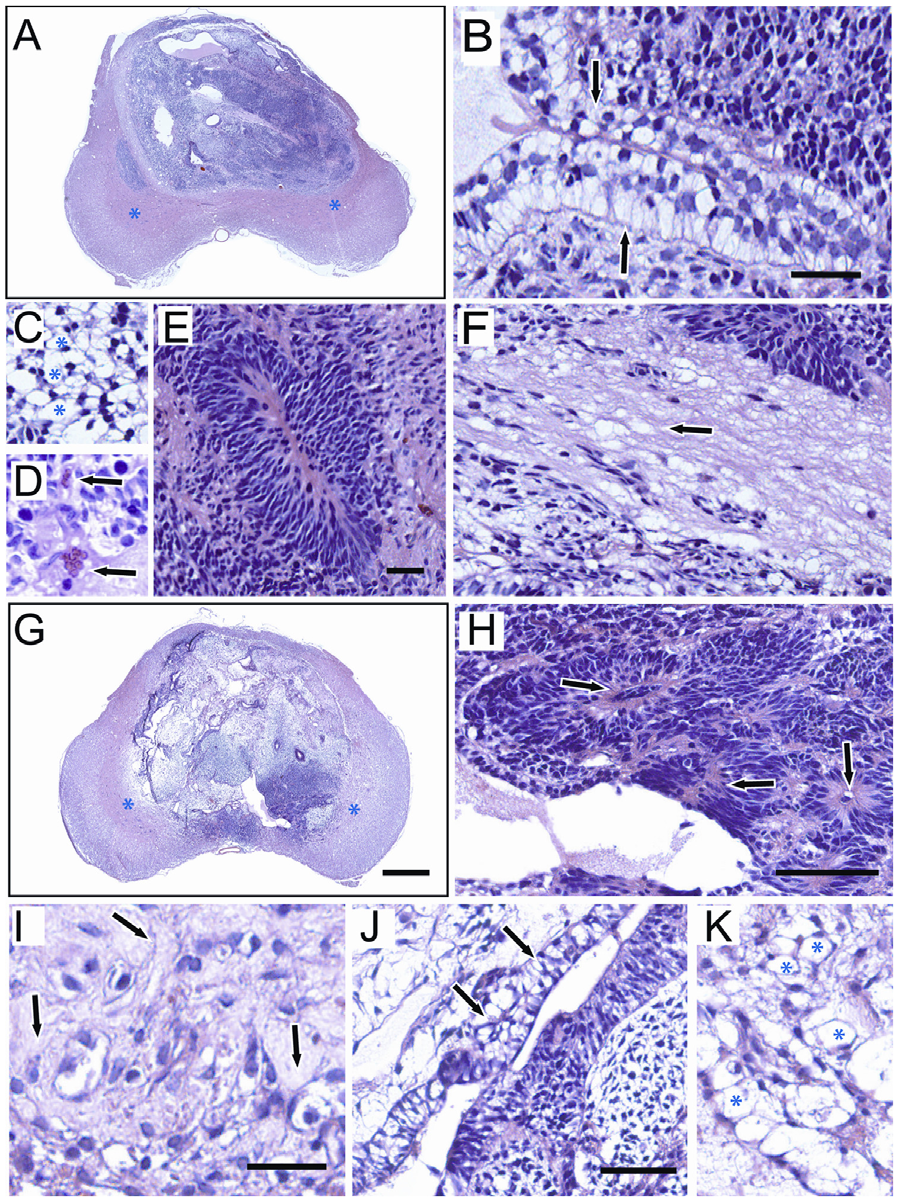
*In vivo* differentiative capacity of hESCs upon extended passaging after sorting with Y-27632. Hematoxylin and Eosin staining of cell-injected spinal cords. Teratomas are from hESCs cultured in feeder-dependent growth conditions passage 21 post-sort (**A–F**) and feeder-independent growth conditions at passage 12 post-sort (**G–K**). Individual germ layers were identified by the presence of ectoderm (neural rosettes) (**E, H**; arrows), endoderm (simple columnar epithelia or goblet cells) (**B, J**; arrows) and mesoderm ((loose connective tissue of early muscle (**F, I** ; arrows), the honey-comb like vacuoles of adipocytes (**C, K**; asterisks) and newly formed blood vessels (**D**) derivatives). Scales bars: (**A,G**): 300 µm; (**H**): 50 µm; (**J, K**): 30 µm; (**B, C, D, E, F and I**): 20 µm).

Additionally, hESCs sorted and treated with Y-27632 were able to differentiate *in vivo* to all three germ layers. Transverse spinal cord sections prepared from animals injected with sorted hESCs grown in feeder-dependent and feeder-independent conditions showed consistent presence of teratomas. This was accompanied by spinal gray matter expansion in injected spinal cord segments. In the majority of sections only a thin rim of residual host tissue in the ventral horn was still recognizable (Fig. 6A, G; blue asterisks). Individual germ layers were identified by the presence of ectoderm (neural rosettes) (Fig. 6E, H; arrows), endoderm (simple columnar epithelia or goblet cells) (Fig. 6B, J; arrows) and mesoderm ((loose connective tissue of early muscle (Fig. 6F, I; arrows), the honey-comb like vacuoles of adipocytes (Fig. 6C, K; asterisks) and newly formed blood vessels (Fig. 6D)) derivatives.

**Figure 7:**
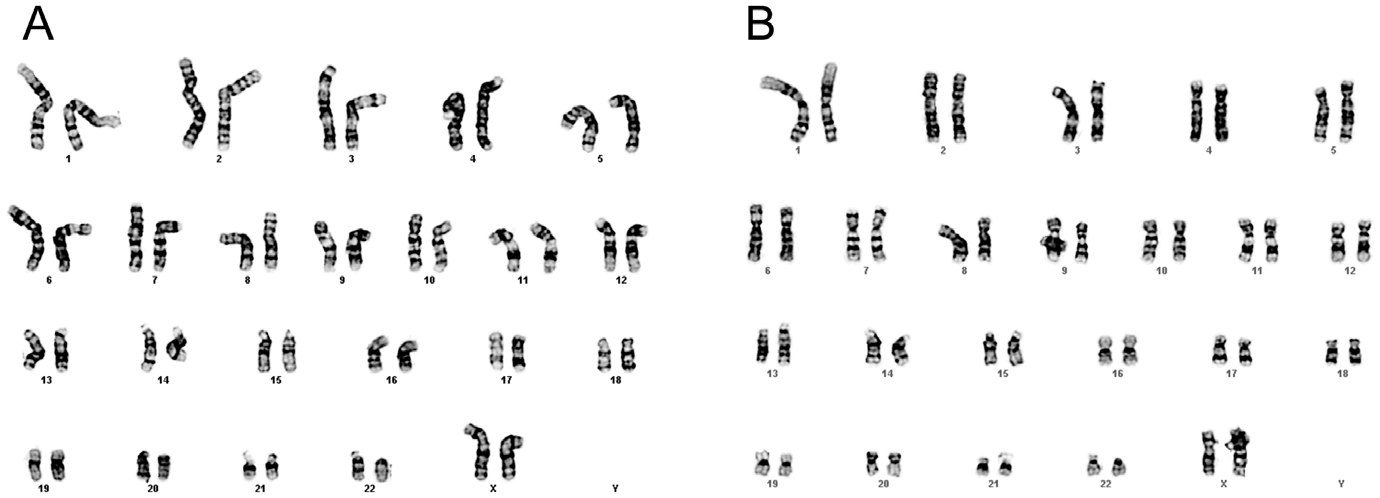
Karyotype analysis of hESCs upon extended passaging after sort with the application of Y-27632. Post-sort karyotype of H9 hESCs grown on **A**) feeders at passage 55 (passage 18 post-sort) with Y-27632 and **B**) feeder-free at passage 50 (passage 21 feeder-free and passage 16 post-sort) with Y-27632.

Sorted cells were passaged for at least 16 passages and were found to exhibit a normal karyotype in feeder-dependent (Fig. 7A) and feeder-independent (Fig. 7B) conditions. Additionally, cells sorted without Y-27632 and cultured long term also exhibited a normal karyotype (Fig. S6). No differences were observed in the karyotypic stability of hESCs that were sorted with and without Y-27632.

## Discussion

We have demonstrated the ability to consistently recover pure populations of hESCs by FACS using cell surface markers and Y-27632. This improved and consistent methodology will aid in the purification of human pluripotent stem cells from cultures that have populations of differentiating cells as well as the removal of various types of feeder layers. Furthermore, as stem cell biology moves towards cell-based therapies, it is crucial that specific cell populations be able to be isolated and purified on the basis of their cell surface signature. Novel markers for sorting of hESC-derived lineage specific populations [32]-[35] and more gentle sorting techniques [36] are being implemented with stem cell populations, but more studies are required. In addition to hESCs, we predict that the addition of Y-27632 would assist in post-sort recovery of various other stem cell types as addition of Y-27632 has enabled numerous other applications for a variety of cells. Y-27632 has been beneficial in post-cryopreservation recovery in not only hESCs [8]–[11] but also hiPSCs [8], [12] as well as non-human primate embryonic stem cells [13] and mesenchymal stem cells [14]. However not all cell types benefit from the application of Y-27632 as fresh and cryopreserved cord blood-derived CD34^+^ hematopoietic stem and progenitor cells were adversely affected upon the application of Y-27632 to cells [37]. Y-27632 has been used recently for sorting for GFP-positive cells in the recovery of viral reprogramming factor-free hiPSCs from Parkinson's patients [38]. Interestingly, in order to examine crypt organoid formation, Y-27632 was added post-sort to clone Lgr5-GFP murine crypts [39]. If Y-27632 is to be added to other stem cell populations and their derivatives post-sort, it will be important to determine the effect Y-27632 may have on the long-term phenotype of cells.

Our results indicate that certain hESC lines are more amenable to FACS than others. This could be due to genotypic differences, cell culturing methodologies or both. HUES-9 had the most robust recovery rates after FACS. Interestingly, this line was adapted to single-cell passaging during its establishment [40]. We hypothesize that one way to improve the percent recovery in additional hESC lines would be to first adapt lines to single-cell passaging prior to FACS. Recent evidence has shown that the addition of Y-27632 to both media and matrix (Matrigel) results in an increase in survival after cryopreservation and passaging as opposed to treatment with Y-27632 in the media alone[41], [42]. It would be interesting to examine if the application of Y-27632 to matrices in addition to media after sorting would benefit hESC lines, such as H7, that are inherently more sensitive to sorting. Pretreatment of hESC with Y-27632 has been reported to aid both single-cell passaging and cryopreservation [7], [12]. We did not observe the same trend with FACS for the hESC lines we examined. We suspect that the additional time and manual titurations required to create a single-cell suspension appropriate for FACS may have caused significant stress that negated its positive effect. One proposed mechanism whereby ROCK inhibition leads to an increase in hESC survival is that the inhibition of Y-27632 increases cell-cell interactions which then allow for aggregates of hESCs to form [28]. While this mechanism would be useful post-FACS, it may be counter productive pre-FACS.

Y-27632 was initially identified while screening for modulators that would increase the low levels of cloning efficiency of hESCs [7], [43]. Previous attempts to clonally derive hESC lines by sorting using forward and side scatter reported an equally low cloning efficiency [23]. It would be of interest to sort hESC to clonality using cells surface makers and our improved methods in cell preparation and sorting and includeY-27632 post-sort to examine if this improves the recovery of sorting hESCs to clonality. It would also be of interest to apply these sorting strategies to sort pure populations of hiPSCs to obtain clonal hiPSC lines or sort fully reprogrammed cells from their intermediates in lieu of having to manually pick colonies [44], [45]. Hence, the advances presented here to successfully sort and recover hESCs using cell surface markers have applications not only in the purification of hESC but also could apply to hiPSC populations. Additionally, this is a step in the direction to advance the use of cell surface markers and flow cytometry to successfully sort derivates of hESCs that has far reaching implications in the field of regenerative medicine.

## Supporting Information

Figure S1 Morphology of hESCs grown on feeders after sorting with application of Y-27632. Bright field images of H7 hESCs on feeders (at passage 38 prior to sorting) at various days after sorting with Y-27632. (*) indicates the day at which cells were passaged after the sort. H7 hESCs that were not treated with Y-27632 after sorting did not yield colonies (data not shown). Scale bars are 100 µm.
(1.47 MB TIF)

Figure S2 Y-27632 pre-treatment of hESCs grown feeder-free and post-sort Y-27632 titration. Day 1 percent recovery post-sort of HUES-9 hESCs after pre-treatment of cells with Y-27632 pre-sort and Y-27632 post-sort titration A–C). Percent recovery equals the number of cells recovered at day 1 post-sort divided by the number of cells plated. A total of three wells were counted for each trial group with error bars representing standard deviation.
(0.23 MB TIF)

Figure S3 Morphology and immunofluorescence of hESCs cultured in feeder and feeder-free conditions upon extended passaging after sorting without the application of Y-27632. A) Bright field image of H9 hESCs on feeders at passage 25 post-sort and day 4 after passaging. B–D) Immunofluorescence labeling of hESCs in feeder growth conditions at passage 25 post-sort. E) Bright field image of hESCs in feeder-free growth conditions at passage 10 post-sort and day 3 after passaging. F–H) Immunofluorescence labeling of hESCs in feeder-free growth conditions at passage 10 post-sort. Scale bars are 100 µm.
(4.56 MB TIF)

Figure S4 Pluripotency marker expression as measured by flow cytometry upon extended passaging after sorting without and with application of Y-27632. Post-sort marker expression of H9 hESCs at A) Passage 14 post-sort with Y-27632 on feeders, B) Passage 13 post-sort without Y-27632 on feeders, C) Passage 13 post-sort feeder-free with Y-27632, and D) Passage 13 post-sort feeder-free without Y-27632.
(0.34 MB TIF)

Figure S5 *In vitro* differentiative capacity of hESCs cultured in feeder and feeder-free conditions upon extended passaging after sorting without Y-27632. Bright field images of A) day 5 embryoid bodies from hESCs on feeders at passage 12 post-sort and B) day 6 embryoid bodies from hESCs feeder-free at passage 12 post-sort. Immunofluorescence of differentiated hESCs shows labeling for mesoderm (B, F) (GATA4 and cTnI), ectoderm (C, G) (Nestin and Sox1), and endoderm (D, H) (FoxA2, Sox17). For feeder conditions (B–D), hESCs are at passage 13 post-sort (B); passage 12 post-sort (C); and passage 15 post-sort (D). For feeder-free conditions (F–H), hESCs are at passage 20 post-sort (F); passage 12 post-sort (G); and passage 19 post-sort (H). Scale bars are 100 µm.
(4.87 MB TIF)

Figure S6 Karyotype analysis of hESCs cultured on feeders and feeder-free conditions upon extended passaging after sort without application of Y-27632. Post-sort karyotype of H9 hESCs grown on A) feeders at passage 54 (passage 17 post-sort) without Y-27632 and B) feeder-free at passage 50 (passage 21 feeder-free and passage 16 post-sort) without Y-27632.
(0.39 MB TIF)

## References

[1] Reubinoff BE, Pera MF, Fong CY, Trounson A, Bongso A (2000). Embryonic stem cell lines from human blastocysts: somatic differentiation in vitro.. Nat Biotechnol.

[2] Thomson JA, Itskovitz-Eldor J, Shapiro SS, Waknitz MA, Swiergiel JJ (1998). Embryonic stem cell lines derived from human blastocysts.. Science.

[3] Hoffman LM, Carpenter MK (2005). Characterization and culture of human embryonic stem cells.. Nat Biotechnol.

[4] Trounson A (2006). The production and directed differentiation of human embryonic stem cells.. Endocr Rev.

[5] Narumiya S, Ishizaki T, Uehata M (2000). Use and properties of ROCK-specific inhibitor Y-27632.. Methods Enzymol.

[6] Riento K, Ridley AJ (2003). Rocks: multifunctional kinases in cell behaviour.. Nat Rev Mol Cell Biol.

[7] Watanabe K, Ueno M, Kamiya D, Nishiyama A, Matsumura M (2007). A ROCK inhibitor permits survival of dissociated human embryonic stem cells.. Nat Biotechnol.

[8] Claassen DA, Desler MM, Rizzino A (2009). ROCK inhibition enhances the recovery and growth of cryopreserved human embryonic stem cells and human induced pluripotent stem cells.. Mol Reprod Dev.

[9] Li X, Krawetz R, Liu S, Meng G, Rancourt DE (2009). ROCK inhibitor improves survival of cryopreserved serum/feeder-free single human embryonic stem cells.. Hum Reprod.

[10] Li X, Meng G, Krawetz R, Liu S, Rancourt DE (2008). The ROCK inhibitor Y-27632 enhances the survival rate of human embryonic stem cells following cryopreservation.. Stem Cells Dev.

[11] Martin-Ibanez R, Unger C, Stromberg A, Baker D, Canals JM (2008). Novel cryopreservation method for dissociated human embryonic stem cells in the presence of a ROCK inhibitor.. Hum Reprod.

[12] Mollamohammadi S, Taei A, Pakzad M, Totonchi M, Seifinejad A (2009). A simple and efficient cryopreservation method for feeder-free dissociated human induced pluripotent stem cells and human embryonic stem cells.. Hum Reprod.

[13] Takehara T, Teramura T, Onodera Y, Kakegawa R, Fukunaga N (2008). Rho-associated kinase inhibitor Y-27632 promotes survival of cynomolgus monkey embryonic stem cells.. Mol Hum Reprod.

[14] Heng BC (2009). Effect of Rho-associated kinase (ROCK) inhibitor Y-27632 on the post-thaw viability of cryopreserved human bone marrow-derived mesenchymal stem cells.. Tissue Cell.

[15] Braam SR, Nauw R, Ward-van Oostwaard D, Mummery C, Passier R Inhibition of ROCK improves survival of human embryonic stem cell-derived cardiomyocytes after dissociation.. Ann N Y Acad Sci.

[16] Koyanagi M, Takahashi J, Arakawa Y, Doi D, Fukuda H (2008). Inhibition of the Rho/ROCK pathway reduces apoptosis during transplantation of embryonic stem cell-derived neural precursors.. J Neurosci Res.

[17] Osakada F, Jin ZB, Hirami Y, Ikeda H, Danjyo T (2009). In vitro differentiation of retinal cells from human pluripotent stem cells by small-molecule induction.. J Cell Sci.

[18] Park IH, Zhao R, West JA, Yabuuchi A, Huo H (2008). Reprogramming of human somatic cells to pluripotency with defined factors.. Nature.

[19] Okumura N, Ueno M, Koizumi N, Sakamoto Y, Hirata K (2009). Enhancement on primate corneal endothelial cell survival in vitro by a ROCK inhibitor.. Invest Ophthalmol Vis Sci.

[20] Lingor P, Tonges L, Pieper N, Bermel C, Barski E (2008). ROCK inhibition and CNTF interact on intrinsic signalling pathways and differentially regulate survival and regeneration in retinal ganglion cells.. Brain.

[21] Adewumi O, Aflatoonian B, Ahrlund-Richter L, Amit M, Andrews PW (2007). Characterization of human embryonic stem cell lines by the International Stem Cell Initiative.. Nat Biotechnol.

[22] Carpenter MK, Rosler E, Rao MS (2003). Characterization and differentiation of human embryonic stem cells.. Cloning Stem Cells.

[23] Sidhu KS, Tuch BE (2006). Derivation of three clones from human embryonic stem cell lines by FACS sorting and their characterization.. Stem Cells Dev.

[24] Eiges R, Schuldiner M, Drukker M, Yanuka O, Itskovitz-Eldor J (2001). Establishment of human embryonic stem cell-transfected clones carrying a marker for undifferentiated cells.. Curr Biol.

[25] Nicholas CR, Gaur M, Wang S, Pera RA, Leavitt AD (2007). A method for single-cell sorting and expansion of genetically modified human embryonic stem cells.. Stem Cells Dev.

[26] Stewart MH, Bosse M, Chadwick K, Menendez P, Bendall SC (2006). Clonal isolation of hESCs reveals heterogeneity within the pluripotent stem cell compartment.. Nat Methods.

[27] Fong CY, Peh GS, Gauthaman K, Bongso A (2009). Separation of SSEA-4 and TRA-1-60 Labelled Undifferentiated Human Embryonic Stem Cells from A Heterogeneous Cell Population Using Magnetic-Activated Cell Sorting (MACS) and Fluorescence-Activated Cell Sorting (FACS).. Stem Cell Rev.

[28] Krawetz RJ, Li X, Rancourt DE (2009). Human embryonic stem cells: caught between a ROCK inhibitor and a hard place.. Bioessays.

[29] D'Amour KA, Agulnick AD, Eliazer S, Kelly OG, Kroon E (2005). Efficient differentiation of human embryonic stem cells to definitive endoderm.. Nat Biotechnol.

[30] Kakinohana O, Cizkova D, Tomori Z, Hedlund E, Marsala S (2004). Region-specific cell grafting into cervical and lumbar spinal cord in rat: a qualitative and quantitative stereological study.. Exp Neurol.

[31] Bajpai R, Lesperance J, Kim M, Terskikh AV (2008). Efficient propagation of single cells Accutase-dissociated human embryonic stem cells.. Mol Reprod Dev.

[32] King FW, Ritner C, Liszewski W, Kwan HC, Pedersen A (2009). Subpopulations of Human Embryonic Stem Cells with Distinct Tissue-Specific Fates Can Be Selected from Pluripotent Cultures.. Stem Cells Dev.

[33] Peh GS, Lang RJ, Pera MF, Hawes SM (2009). CD133 expression by neural progenitors derived from human embryonic stem cells and its use for their prospective isolation.. Stem Cells Dev.

[34] Sundberg M, Jansson L, Ketolainen J, Pihlajamaki H, Suuronen R (2009). CD marker expression profiles of human embryonic stem cells and their neural derivatives, determined using flow-cytometric analysis, reveal a novel CD marker for exclusion of pluripotent stem cells.. Stem Cell Res.

[35] Zhao D, Chen S, Cai J, Guo Y, Song Z (2009). Derivation and characterization of hepatic progenitor cells from human embryonic stem cells.. PLoS One.

[36] Pruszak J, Sonntag KC, Aung MH, Sanchez-Pernaute R, Isacson O (2007). Markers and methods for cell sorting of human embryonic stem cell-derived neural cell populations.. Stem Cells.

[37] Bueno C, Montes R, Menendez P The ROCK Inhibitor Y-27632 Negatively Affects the Expansion/Survival of Both Fresh and Cryopreserved Cord Blood-Derived CD34+ Hematopoietic Progenitor Cells: Y-27632 negatively affects the expansion/survival of CD34+HSPCs.. Stem Cell Rev.

[38] Soldner F, Hockemeyer D, Beard C, Gao Q, Bell GW (2009). Parkinson's disease patient-derived induced pluripotent stem cells free of viral reprogramming factors.. Cell.

[39] Sato T, Vries RG, Snippert HJ, van de Wetering M, Barker N (2009). Single Lgr5 stem cells build crypt-villus structures in vitro without a mesenchymal niche.. Nature.

[40] Cowan CA, Klimanskaya I, McMahon J, Atienza J, Witmyer J (2004). Derivation of embryonic stem-cell lines from human blastocysts.. N Engl J Med.

[41] Baharvand H, Salekdeh GH, Taei A, Mollamohammadi S An efficient and easy-to-use cryopreservation protocol for human ES and iPS cells.. Nat Protoc.

[42] Pakzad M, Totonchi M, Taei A, Seifinejad A, Hassani SN Presence of a ROCK inhibitor in extracellular matrix supports more undifferentiated growth of feeder-free human embryonic and induced pluripotent stem cells upon passaging.. Stem Cell Rev.

[43] Amit M, Carpenter MK, Inokuma MS, Chiu CP, Harris CP (2000). Clonally derived human embryonic stem cell lines maintain pluripotency and proliferative potential for prolonged periods of culture.. Dev Biol.

[44] Lowry WE, Richter L, Yachechko R, Pyle AD, Tchieu J (2008). Generation of human induced pluripotent stem cells from dermal fibroblasts.. Proc Natl Acad Sci U S A.

[45] Yamanaka S (2009). Elite and stochastic models for induced pluripotent stem cell generation.. Nature.

