# Supplementary figures and images for: The ROCK Inhibitor Y-27632 Improves Recovery of Human Embryonic Stem Cells after Fluorescence-Activated Cell Sorting with Multiple Cell Surface Markers
Source: PLoS One. 2010 Aug 13;5(8):e12148. doi: 10.1371/journal.pone.0012148 (PMC2921395; doi:10.1371/journal.pone.0012148)

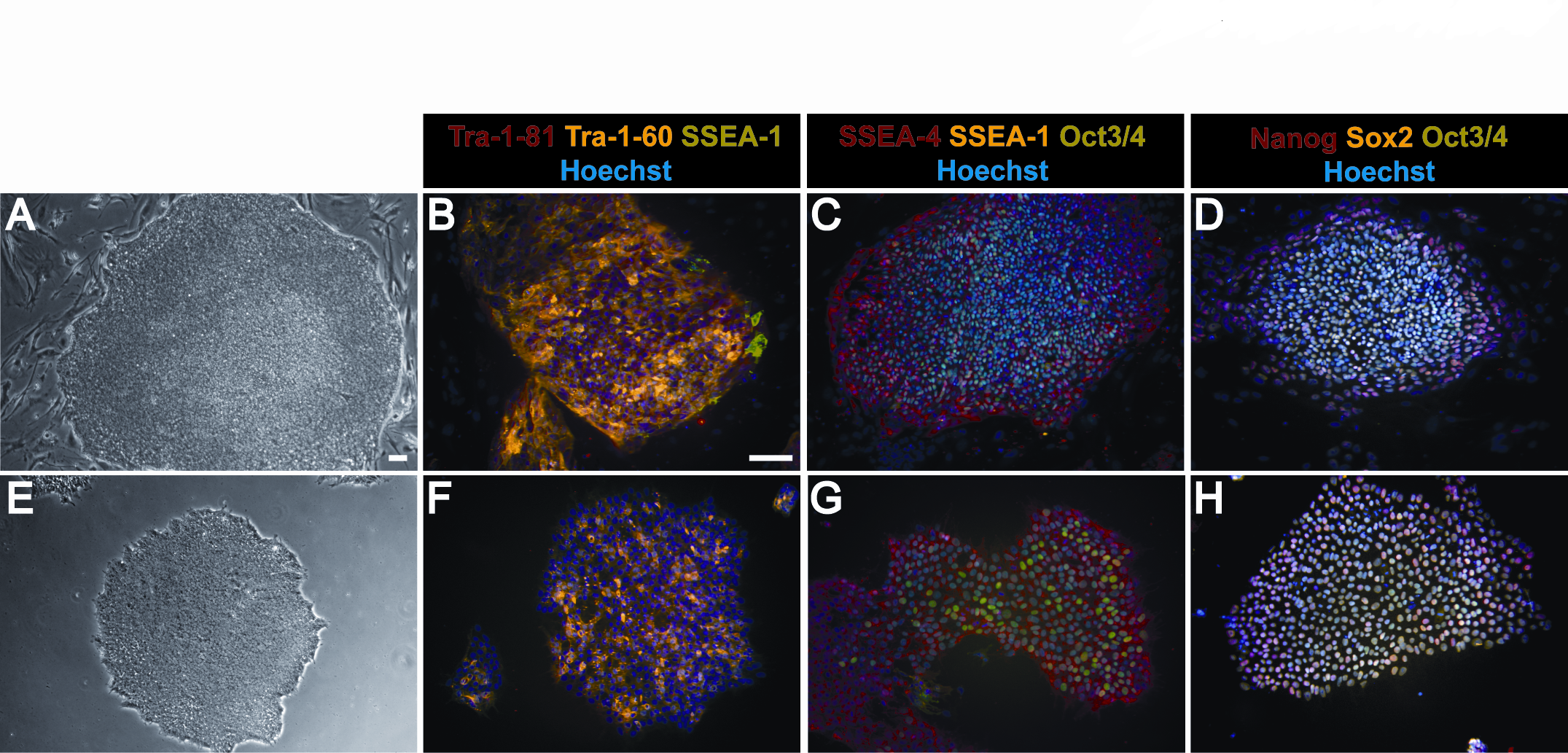

Supplement: Figure S3 — Morphology and immunofluorescence of hESCs cultured in feeder and feeder-free conditions upon extended passaging after sorting without the application of Y-27632. A) Bright field image of H9 hESCs on feeders at passage 25 post-sort and day 4 after passaging. B–D) Immunofluorescence labeling of hESCs in feeder growth conditions at passage 25 post-sort. E) Bright field image of hESCs in feeder-free growth conditions at passage 10 post-sort and day 3 after passaging. F–H) Immunofluorescence labeling of hESCs in feeder-free growth conditions at passage 10 post-sort. Scale bars are 100 µm. (4.56 MB TIF) [file pone.0012148.s003.tif]

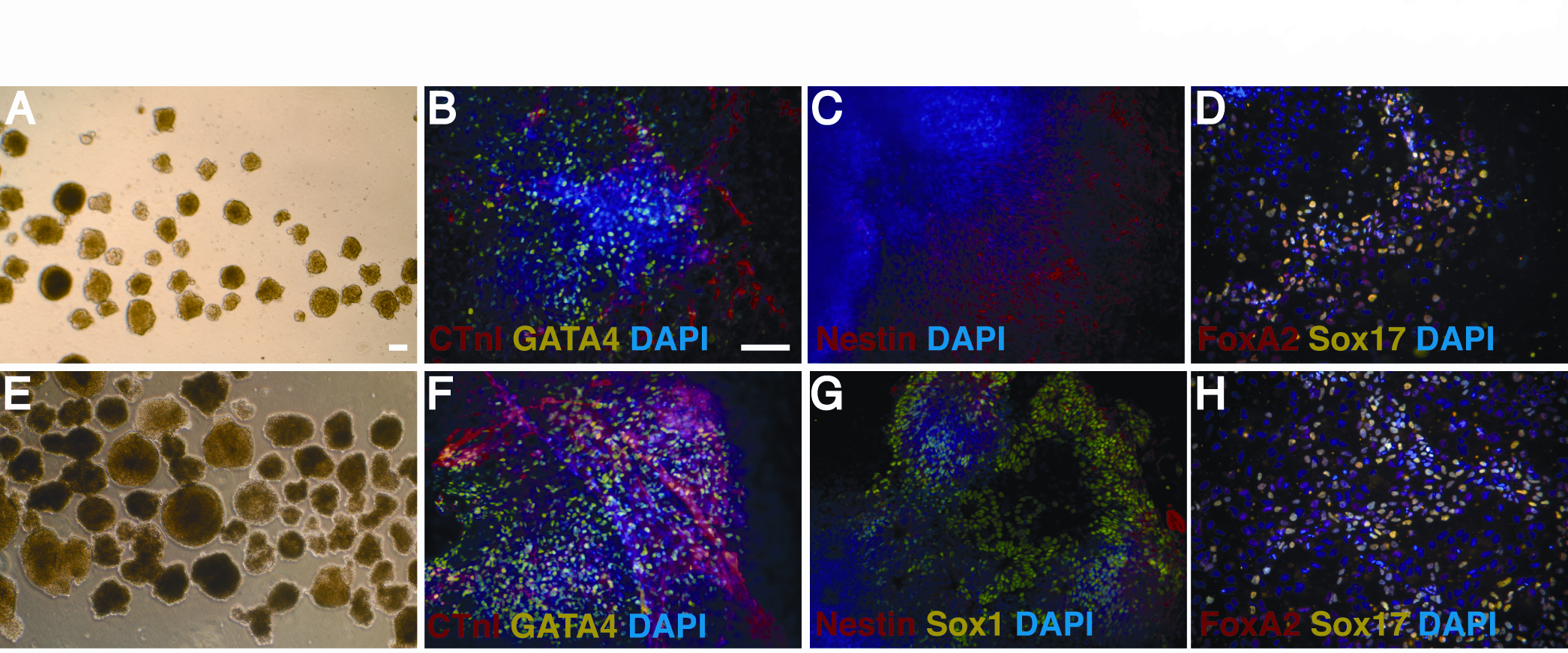

Supplement: Figure S5 — In vitro differentiative capacity of hESCs cultured in feeder and feeder-free conditions upon extended passaging after sorting without Y-27632. Bright field images of A) day 5 embryoid bodies from hESCs on feeders at passage 12 post-sort and B) day 6 embryoid bodies from hESCs feeder-free at passage 12 post-sort. Immunofluorescence of differentiated hESCs shows labeling for mesoderm (B, F) (GATA4 and cTnI), ectoderm (C, G) (Nestin and Sox1), and endoderm (D, H) (FoxA2, Sox17). For feeder conditions (B–D), hESCs are at passage 13 post-sort (B); passage 12 post-sort (C); and passage 15 post-sort (D). For feeder-free conditions (F–H), hESCs are at passage 20 post-sort (F); passage 12 post-sort (G); and passage 19 post-sort (H). Scale bars are 100 µm. (4.87 MB TIF) [file pone.0012148.s005.tif]
